# Testing the feasibility of a sustainable preschool obesity prevention approach: a mixed-methods service evaluation of a volunteer-led HENRY programme

**DOI:** 10.1186/s12889-020-10031-w

**Published:** 2021-01-06

**Authors:** Neil Howlett, Kim P. J. Roberts, Di Swanston, Laurel D. Edmunds, Thomas A. Willis

**Affiliations:** 1 Department of Psychology, Sport, and Geography, University of Hertfordshire, College Lane, Hatfield, Herts AL10 9AB UK; 2 HENRY, 8 Elm Place, Old Witney Road, Oxfordshire, OX29 4BD UK; 3 Radcliffe Department of Medicine, Medical Sciences Division, University of Oxford, John Radcliffe Hospital, Oxford, OX3 9DU UK; 4 Leeds Institute of Health Sciences, University of Leeds, Leeds, LS2 9NL UK

**Keywords:** HENRY, Preschool obesity prevention, Behaviour change, Parent-focused, Volunteers, Healthy eating, Mixed-methods

## Abstract

**Background:**

Over the last 10 years HENRY has been working to reduce and prevent child obesity by training health and early years professionals to deliver its evidence-based programme to parents. The aim and unique contribution of this study was to evaluate whether training volunteers to deliver this programme on a one-to-one basis was feasible.

**Methods:**

Mixed-methods service evaluation with parent-reported pre- and post-programme outcomes and focus groups conducted with parents and volunteer facilitators. The programme consisted of 8 one-to-one sessions delivered weekly by volunteers (*n* = 18) to build food and activity-related knowledge, skills, and understanding, and improve parenting efficacy, and parent and child eating and physical activity. Programmes took place at parent’s (*n* = 69) home or local community venues in four London boroughs, United Kingdom. Parent-reported parenting efficacy, emotional wellbeing, eating, and physical activity data were captured, alongside parent ratings of the programme and volunteer ratings of the training. Parent and volunteer focus groups explored involvement, expectations, and experiences of the programme, training and delivery, feedback, and impact.

**Results:**

Parents were mostly female, had varied ethnic backgrounds, and were often not working but well educated. There were statistically significant improvements of a medium-to-large size in parent and child emotional wellbeing, parenting efficacy, fruit and vegetable consumption, family eating and food purchasing behaviours. Parent ratings of the programme were positive and qualitative data highlighted the holistic nature of the programme, which focused on more than just food, and the relationships with volunteers as key facets. Volunteers were also mostly female, had varied ethnic backgrounds, and were often well educated, but more likely to be employed than parents. Volunteers rated the training and delivery as useful in enabling them to deliver the programme confidently and for their own wellbeing. Despite finding some sessions challenging emotionally, volunteers reported positive family lifestyle improvements by parents and children and that the experience would be useful for future employment.

**Conclusions:**

It is feasible to recruit and train volunteers to deliver a structured preschool obesity prevention programme, which parents considered acceptable and enjoyable, with preliminary reports of parent and child benefits.

**Supplementary Information:**

The online version contains supplementary material available at 10.1186/s12889-020-10031-w.

## Background

There is a clear need for interventions to help families of infants, toddlers, and preschool children adopt a healthier lifestyle: in England, by the age of 4–5 years almost 25% of children are overweight, rising to a third by age 10–11 years [1]. Moreover, only 10% of children aged 2–4 years achieve recommended daily physical activity levels [2]. The food intake of infants and toddlers aged 6–18 months is associated with maternal intake on a range of foods [3], and research has shown that mothers of young children (aged 3–30 months) are amenable to holistic parent-targeted approaches to infant and toddler health in the form of community-based interventions [4]. Furthermore, for infants and toddlers aged 0–2 years old, interventions can be effective in promoting behaviours such as healthier eating but few studies measure longer-term impact [5]. When the age group is widened to 0–5 years old, overweight and obesity prevention interventions show a lack of consistent evidence for improvements in weight-related outcomes [6]. Only three of 17 interventions targeting 0–5 year olds were found to be effective, and two of these incorporated parental involvement. More positive outcomes have been found from programmes targeting parents to improve nutrition behaviour, knowledge, and efficacy.

Parents of pre-school children (aged 3–4 years old) describe challenges in assessing portion sizes for both themselves and their children, providing adequate options for a range of food preferences within the family, and maintaining consistent rules around eating behaviour more generally [7]. Consequently, although interventions for 2–5 year olds can work, they are more effective when incorporating greater parental involvement and skills training [5]. Obesity prevention programmes in 2–6 year olds also find better outcomes with parent-focused training and individual support [8]. A review of obesity prevention studies targeted at 0–6 year olds, found that the majority produced significant positive effects on both eating (72%) and physical activity (77%) [9] behaviour. Even in these often complex interventions, targeted at multiple behaviours and levels of the system, effectiveness is more likely with strong parental engagement [9]. Additionally, if obesity prevention programmes achieve relatively small sustainable effects for outcomes such as BMI in 2–5 year olds, the cost saving and health benefits could be considerable [10].

The UK charity HENRY (Health, Exercise, Nutrition for the Really Young) has a strong evidence base in providing multi-level behaviour change programmes for parents with babies, toddlers, and preschool children (0–5 years old). HENRY offers both effective training for health and early years professionals (e.g. Sure Start Centre staff) and delivery of family programmes to help parents achieve healthier outcomes for themselves and their preschool children. Research has demonstrated the impact of HENRY training on individuals, in terms of both their professional and personal lives. For example, training was associated with increased confidence and knowledge in both understanding the factors associated with child obesity, and having challenging conversations with parents about weight and lifestyle issues [11–14]. These benefits have been reported up to 4 years post-training [14]. In terms of personal impact, HENRY-trained staff report increased knowledge of appropriate portion sizes and establishing more family mealtimes [12, 14]. Over 12,000 health and early years practitioners have participated in HENRY training. Meanwhile, parents of children aged 0–5 years old, who have completed the weekly face-to-face programme, consistently report improvements in parenting efficacy, emotional wellbeing, and family eating behaviours, and the importance of being listened to and encouraged to develop their own ideas [15, 16]. There are indications that these positive emotional and behavioural changes are sustained months after the programme ends [13].

Parent-focused programmes can form part of successful population-level initiatives to tackle obesity in 4–5 year olds [17]. Who delivers these programmes and how successfully they are embedded within local communities are important factors. Trained non-professional staff (also referred to as community health workers, or lay health advisors) have successfully delivered a range of health-related interventions, e.g. increasing immunization uptake, or reducing BMI, blood pressure and even child mortality [18–20]. The UK Government has advocated the use of volunteers to deliver interventions in their own communities to foster greater community engagement and social capital [21]. Observational evidence suggests that volunteering has benefits for volunteer wellbeing, including increased life satisfaction and reduced depression and mortality [22].

Research is required into the potential feasibility of volunteer-delivered health promotion interventions for parents with children from 0 to 5 years old, whether volunteers can be recruited from the same ethnic and socioeconomic backgrounds as parents, and to explore the experiences of parents and volunteers involved in these programmes. HENRY has a proven track record of training health and early years professionals to deliver parent-directed programmes but the feasibility of the approach has not been tested with volunteer-led delivery. This study adopted a mixed methods approach to address the following aims:

- To explore whether parents can achieve reported improvements in parenting, eating behaviour and attitudes, physical activity, and emotional wellbeing, with programmes delivered by volunteers
- To explore feasibility issues in terms of parent recruitment (e.g. demographics), ratings of the programme, and qualitative feedback on their experiences of the programme
- To explore feasibility issues in terms of volunteer recruitment (e.g. demographics), and ratings and qualitative feedback on their training and delivery experiences

## Method

This study was a mixed-methods evaluation, with parent-reported pre- and post-programme outcomes and focus groups providing qualitative feedback from parents and volunteer facilitators. The evaluation explored the feasibility of delivering an existing evidence-based obesity prevention programme for parents and preschool children, by trained volunteers. The programme comprised 8 one-to-one sessions delivered by volunteers weekly, but with some flexibility in this timeline to accommodate other commitments or illness. The aims of the programme were to improve parents’ food and activity-related knowledge, skills, and understanding, parenting efficacy, parent and infant emotional wellbeing, eating, and physical activity.

### Parents

Eligible parents had to have or care for a child up to 5 years old. Parents were recruited via referrals from local health and early years professionals, as well as direct community engagement activities.

### HENRY programme

HENRY’s one-to-one programme, *Healthy Families Right from the Start,* was adapted, in terms of delivery style but not content, from HENRY’s group programme for parents, which has been covered in detail elsewhere [13, 15]. Prior to this study, one-to-one programme delivery had been tested and refined over a 2-year period and delivered by paid health and early years practitioners. HENRY trained Family Lives volunteers to deliver the structured programme; Family Lives (www.familylives.org.uk) is a UK charity supporting volunteers to deliver parent-to-parent support. Volunteers worked in partnership with individual families to build knowledge, skills, and understanding of a healthy start in life in five key areas: parenting; eating habits and attitudes to feeding; healthy eating; physical activity; emotional wellbeing.

The programme comprised an integrated approach based on the Family Partnership Model [23], motivational interviewing, and solution-focused support [24]. This approach emphasized the parent-facilitator relationship and enabled families to develop the confidence and motivation to make positive changes by creating a supportive environment, building readiness for change, and developing self-efficacy [24]. The programme was delivered over 8 × 1 h sessions, either at the parent’s home or a local community venue, in four boroughs of London, UK: Haringey; Hackney; Westminster; Ealing.

### Materials

Families received the HENRY workbook containing eight main sections, which combined practical activities with pictorial information and covered the following areas: emotional wellbeing; parent-child relationships; early feeding; eating healthily for the whole family; family mealtimes; physical activity; understanding and responding to children’s feelings and setting limits. Each session included reflection and goal setting activities. The volunteers received a practitioner guide which supported programme fidelity by providing a structured framework of a comprehensive session plan for each week of the programme, with step-by-step directions of how to lead the activities and discussions. Alongside the session plans, the guide provided detailed notes on the skills and approach to be used when delivering sessions. This enabled volunteers to revisit and reinforce what they learned on the training.

### Volunteer training

Volunteers were recruited from local communities in an attempt to represent different cultural and ethnic groups accessing preventive health services. The main requirements for volunteers were: experience of parenting, being in a parenting role, or demonstrable experience of working with parents or children; willingness to complete training, supervision, comply with HENRY policy and procedures, and commit to the role for 12 months. Volunteers completed 5 days’ training to equip them with the confidence, skills and knowledge to deliver the intervention. During the training, volunteers practised delivering sections of the practitioner guide, with trainers providing support and feedback, ensuring confidence using the HENRY approach. The cost of training each volunteer was approximately £300, which includes trainer fees, venue hire, training materials and refreshments.

The content of the one-to-one programme remained the same as the group programme. However, the style of delivery was adapted to maintain the HENRY approach of interactive, experiential learning when working with an individual rather than a group. This included: replacing small group and pairs activities with facilitated individual reflection and exploration; using workbook activities and short video excerpts as a springboard for discussion between parent and volunteer; use of questioning skills to help a parent ‘discover’ the information in the workbook rather than the volunteer simply explaining the information; creating a choice of activities so that volunteers could tailor delivery to individual learning styles; where possible, harnessing the added value of delivering the programme in the home setting e.g. looking at the labels on food items in the family’s fridge/food cupboard. Travel expenses for training and programme delivery were reimbursed.

To maximise fidelity volunteers were supported and supervised by project staff. This included co-delivery of the first session with newly trained volunteers to enable observation and mentoring, feedback on structured review forms after each session, and regular practice development groups with other volunteers. Structured review forms, evaluating how each session had gone, including challenges, were emailed to project staff who would provide feedback via email or, if issues had arisen, a telephone call. Practice development groups provided opportunities to practise skills and delivering session content as well as sharing challenges and successes. The cost of delivering the programme with one family was approximately £215, which includes coordinator time to support the volunteer and parent resources. The volunteer support time per family was approximately 9 h, which included co-delivery of the first session, weekly calls, and supervision.

### Parent measures

Measures were largely based on previous evaluations [15]. Most measures were completed before the first session (pre-programme) and at the end of session 8 (post-programme). The exceptions were attendance and programme satisfaction (post-programme only), and stepping stones (pre-programme, session 5, post-programme). The outcomes were post-programme changes in parenting efficacy, eating behaviour and attitudes, physical activity, and emotional wellbeing. Where appropriate the internal consistency of measures was calculated and generally ranged from acceptable to good [25].

#### Attendance and programme satisfaction

Weekly attendance was monitored. Parents were asked how useful they found the sessions (1 ‘*Not useful at all*’ – 5 ‘*Very useful*’) and whether they would recommend the programme to other families (1 ‘*No*’ – 5 ‘*Definitely*’).

#### Stepping stones

In sessions 1, 5, and 8, parents completed an activity (‘Stepping stones’) where they considered how healthy was their family’s lifestyle (1 ‘*Not very healthy*’ – 10 ‘*Perfect*’), and how well they were doing in giving their children a healthy start in life (1 ‘*Not very well*’ – 10 ‘*Terrific*’).

#### Parenting efficacy and ability to set limits

Parenting efficacy was assessed with the five-item Parenting Self-Agency Measure, which showed acceptable internal consistency with a range of mothers in the original study [26] and further acceptable internal consistency and test-retest reliability in a large sample of mothers of infants and school age children [27]. The five items were prefaced by asking parents how confident they were from 1 ‘*Never*’ to 5 ‘*Always*’. An example was ‘I feel sure of myself as a mother/father’ (internal consistency in this sample; pre *a* = .83, post *a* = .78). The five items measuring ability to set limits were developed for previous evaluations [13, 15] and showed average to good internal consistency. Ability to set limits was captured in relation to five areas (e.g. mealtimes, bedtime) on a 5-point scale (1 ‘*Not well*’ - 5 ‘*Very well*’) (internal consistency in this sample; pre *a* = .76, post *a* = .74).

#### Emotional wellbeing of parents and children

Five questions examined how often parents experienced stress, time for themselves, support, feelings of isolation, and feelings of happiness and were based on a previous evaluation with acceptable internal consistency [15]. The five items were measured using a 4-point scale (1 ‘*Never/hardly ever*’ - 4 ‘*Very often*’) (internal consistency in this sample; pre *a* = .69, post *a* = .61). Child wellbeing was measured using two subscales of the TNO-AZL Preschool Children Quality of Life questionnaire [28]. Average to good internal consistency was found in the original study for the positive mood and liveliness subscales in two different populations of parents with young children (1–5 years old) [28], and in a later study with nearly 500 mothers of infants and preschool children with good test-retest reliability [29]. Parents were asked three items (good spirits; cheerful; happy) about their child’s positive mood (internal consistency in this sample; pre *a* = .72. post *a* = .83) and liveliness (energetic; active; lively; internal consistency in this sample; pre *a* = .77, post - all parents answered ‘often’ on the lively item) in the last month on a 3-point scale (1 ‘*Never*’ - 3 ‘*Often*’).

#### Eating behaviours

Eating behaviours were assessed using six items based on the modified Family Eating and Activity Habits Questionnaire (e.g. sitting to eat with others, eating standing up), which showed good internal consistency and test-retest reliability in the original measure [30]. Two personal eating items also asked about ‘stopping eating when you have had enough, even if food is left’, and ‘choosing to eat meals that are healthy’. These items were analysed individually after poor internal consistency in this sample as a combined scale, pre *a* = .58; post *a* = .56. A further question was adapted from the original measure and has been used in a previous evaluation [15], with parents asked to circle which snacks they normally have at home (e.g. crisps, popcorn, biscuits).

The remaining questions were all unique to this evaluation and did not come from previously validated measures. Parents then reported how often children eat with an adult at home across five potential time points (breakfast, morning snack, midday meal, afternoon snack, evening meal; internal consistency, pre *a* = .87; post *a* = .81) (1 ‘*Never*’ - 5 ‘*Always*’). This was followed by three individual items about set eating times with preschool children on a 5-point scale (1 ‘*Never*’ - 5 ‘*Always*’) (e.g. How often do you allow them to eat whatever they want?). Finally, five individual items explored how often parents adopted certain approaches to food purchasing and preparation (1 ‘*Never*’ to 5 ‘*Very often*’) (e.g. ‘Read the information labels on food packaging’).

#### Food intake

Habitual family food intake was reported both in relation to the parent and their child (ren) and was unique to this evaluation. Respondents indicated how often, on a scale from 0 to 8+ times per day, they consumed: fruit & vegetables; water; sweetened/fizzy drinks; high fat or high sugar foods.

#### Physical activity and screen time

Physical activity and screen time were measured in line with previous evaluations [13, 15]. Parents reported for how long each day they and their child (ren) participated in physical activity, ranging on a 5-point scale from ‘*None*’ to ‘*More than 1 hour*’ for parents. Child physical activity was specified for children who were able to walk or for those not yet walking. Both were measured on a 5-point scale from ‘*None*’ to ‘*More than 3 hours*’. Screen time per day was assessed for children aged 0–2 years, 3–5 years, and adults respectively, on a 5-point scale from ‘*None*’ to ‘*More than 3 h*’.

### Volunteer measures

Volunteers were asked to evaluate the following: the training (1 ‘*Very poor*’ to 5 ‘*Very good*’); their level of confidence post-training (1 ‘*Not at all confident*’ to 4 ‘*Very confident*’); whether they would recommend the training to other volunteers (1 ‘*No*’ to 5 ‘*Definitely*’); post-training support and supervision, and how helpful they thought the sessions were for parents (1 ‘*Not at all helpful*’ to 4 ‘*Very helpful*’); how happy they were about their delivery of the programme (1 ‘*Not at all happy*’ to 4 ‘*Very happy*’)*.*

### Focus groups

Focus groups explored the experiences of those taking part in, and delivering, the HENRY programme, what ideas might be most useful to incorporate into any further parenting/obesity prevention programmes, and the impact of the programme being delivered by volunteers. Participants were based in Ealing (separate volunteer and parent groups) and Haringey (parent group only), London. Four volunteers and 12 parents (five from Ealing and seven from Haringey) met at convenient settings used for community-based interventions (e.g. Enterprise centre, Haringey). All parents were female and from ethnically diverse backgrounds (3 south Asian, 2 Afro-Caribbean, 7 Caucasian). Participants were self-selected, responding to requests from local coordinators by email, and so the views presented here may or may not represent those of all participants. The parents had completed programmes approximately 3 months before the focus groups took place. The volunteers had to have delivered at least one full programme to be eligible for the focus group.

A topic guide for the focus groups, based on similar evaluations, was prepared with semi-structured questions providing the opportunity for parents and volunteers to offer their thoughts on their experiences (see Supplementary materials). Topics for parents and volunteers covered: early involvement and expectations of the programme; experience of the programme; volunteer facilitators (parents only); training and delivery (volunteers only); information provided; feedback; and improvements. Focus groups were conducted by a female qualitative researcher (LE, PhD) experienced in evaluating similar programmes, who was completely independent to the local delivery team and participants, to allow unbiased opinions to be obtained from volunteers and parents. Only the researcher and participants were present for the focus groups. The aim was to cover the topics rather than to ask a series of questions. The focus groups ended when the topics were discussed, and participants felt they had said all they wanted to. The focus groups were conducted between January and June 2017 and their average time was 47 min. Formal field notes were not part of the evaluation and transcripts were not returned to participants as the only point of contact was the HENRY facilitator.

### Data analyses

All quantitative analysis was completed on SPSS statistical software (version 23, IBM United Kingdom Limited, Portsmouth, UK; 2015). Food frequency data were analysed using repeated measures t-tests (interval level data). Parental emotional wellbeing items were recoded and scored in the same direction. Parenting efficacy, setting limits, parent and child emotional wellbeing, eating behaviours, physical activity, screen time, and stepping stone measures were analysed using paired-sample Wilcoxon signed rank tests (ordinal level data). Data were reanalysed for the number of adults and children reaching daily nutritional and physical activity recommendations (i.e. exercise for at least 30 mins for adults and 3 hours for children under five years) [31]. These categories were analysed using McNemar’s test (for nominal level data). Due to the high number of tests being conducted, a significance level of *p* < .01 was applied throughout to reduce the chance of a type 1 error [15].

Focus groups were audio recorded with participants’ consent and transcribed verbatim. The transcripts were analysed through a process of constant comparison which allowed for the interpretation of data and identification of key themes. This approach is underpinned by Grounded Theory, providing a framework to explore participants’ experiences [32]. Data were collected and analysed concurrently by LE using an iterative written approach. The aim was to identify main themes (often linked to the topic guide) and their associated sub-themes (based on the range of participants’ experiences) from the data. Within a relatively homogenous group (i.e. parents who had completed the HENRY programme), data saturation is usually reached with 40 interviews [33] and so all completers were invited to take part in an effort to meet this criterion.

## Results

### Parent recruitment characteristics, ratings, and outcomes

The majority of the 69 parents were female (97%) and aged 34 years on average (range 18–47 years). Parents came from a range of ethnic backgrounds with 58% in Black, Asian and minority ethnic categories, of which African (13%) and Indian (12%) were the most prevalent. The majority (52%) of parents were not working, the most prevalent category for both maternal (33%) and paternal education (28%) was a Bachelor’s degree, and the most frequently reported household income was low (under £25,000). On average, parents had just over one child per household, with 14 children under 1 year of age, 14 children 1–2 years old, 19 children 2–3 years old, 15 children 3–4 years old, 22 children 4–5 years old, and 8 children 5 years old. Average attendance was 7.2 (SD = 1.9) sessions out of 8. Parents rated the programme as very useful (84%) and would definitely recommend the programme to other families (83%).

Significant improvements were seen in parent-reported parenting efficacy, ability to set limits, parental emotional wellbeing, child mood and liveliness, and family eating behaviours including eating with others, not eating while watching TV, not eating when angry/bored/low, and eating healthy meals (see Table 1). Improvements were also seen in parental awareness of portion sizes and trying new recipes. Effect sizes across these changes were predominantly in the medium-to-large range. These reported improvements did not extend to the kind of snacks kept in the house with no changes post-intervention on any snacking items. From the stepping stones measure, parent-reported lifestyle scores improved from pre-programme (M = 5.72, SD = 1.61) to session 5 (M = 7.08, SD = 1.27) and then again from session 5 to post-programme (M = 8.30, SD = 1.27) (all *p* < .001). Child healthy start scores improved from pre-programme (M = 6.95, SD = 1.88) to session 5 (M = 7.58, SD = 1.50), and then again from session 5 to post-programme (M = 8.80, SD = 1.01) (all *p* < .001).

**Table 1:** Pre and post-programme means (SD) for reported parent and infant outcomes (*N* = 69)

Item/Scale	Score range	Pre	Post	Test result ***p***-value (effect size)
**Parenting efficacy**	5–25	20.54 (3.17)	22.06 (2.18)	< .001 (r = .35)
**Setting limits**	5–25	18.77 (4.15)	21.98 (2.78)	< .001 (r = .47)
**Parental emotional wellbeing**	4–16	12.98 (3.27)	15.34 (2.32)	< .001 (r = .43)
**Children eating with an adult**	5–25	17.58 (5.63)	18.77 (5.06)	.003 (r = .26)
**Children emotional wellbeing**				
Positive Mood	3–9	8.53 (.88)	8.88 (.52)	.001 (r = .29)
Liveliness	3–9	8.71 (.77)	8.97 (.17)	.010 (r = .23)
**Family eating behaviours**				
Eat with others	1–5	3.75 (1.09)	4.14 (.74)	.005 (r = .25)
Eat standing up	1–5	1.98 (1.20)	1.64 (.86)	.027 (r = .19)
Eat straight from pan	1–5	1.71 (1.12)	1.40 (.70)	.029 (r = .19)
Eat while watching TV	1–5	3.04 (1.07)	2.61 (1.11)	.002 (r = .27)
Stop eating when full	1–5	3.35 (1.26)	3.51 (1.05)	.598 (r = .05)
Eat when angry/bored/low	1–5	2.62 (1.15)	2.13 (1.01)	.001 (r = .28)
Eat takeaway food	1–5	2.66 (.92)	2.51 (.83)	.053 (r = .17)
Eat healthy meals	1–5	3.97 (.77)	4.31 (.72)	.004 (r = .24)
**Child eating behaviour**				
Eat when they want	1–5	3.13 (.98)	2.74 (.98)	.054 (r = .20)
Eat what they want	1–5	2.85 (1.17)	2.42 (1.01)	.019 (r = .25)
Eating at set times	1–5	3.82 (1.16)	4.14 (.94)	.065 (r = .19)
**Food purchasing/preparation**				
Think about portion sizes	1–5	3.54 (1.19)	4.19 (.81)	< .001 (r = .32)
Read labels on food packaging	1–5	3.73 (1.25)	4.01 (.99)	.055 (r = .17)
Balance food groups	1–5	4.03 (.84)	4.30 (.81)	.108 (r = .14)
Worry about underfeeding	1–5	2.87 (1.41)	2.74 (1.38)	.327 (r = .09)
Try new recipes	1–5	3.47 (.94)	4.03 (.80)	< .001 (r = .37)

Total scales in bold and single items in normal font. Single items reported when analysis did not support a total scale score. *P*-value results represent pre-post time effects using paired-sample Wilcoxon signed rank tests (criteria for significance, *p* < .01). Effect size represents Pearsons r (criteria: 0.1 = small; 0.3 = medium; 0.5 = large)

The number of times per day parents reported that they and their children were eating fruit and vegetables increased significantly for both parents and children, but the reported proportion of parents and children consuming at least five portions per day did not increase (see Table 2). The number of reported times per day children drank water also increased significantly. For activity-related outcomes neither the reported percentage of parents completing at least 30 min per day, nor children completing at least 3 hours per day, in line with the recommended amount of daily activity, improved. Also, parents and children aged 0–2 years old showed no changes in reported screen time, whereas children aged 3–5 years old showed significant reductions in screen time (Z = − 2.68, *p* = .007).

**Table 2:** Reported food frequency and physical activity data for parents and child (ren) (*N* = 69)

Item/Scale	Parent Score			Test result	Child Score			Test result
	Times per day	Pre	Post	***p***-value (effect size)	Times per day	Pre	Post	***p***-value (effect size)
**Food intake**								
Fruit and vegetables	0–8+	3.25 (1.65)	4.13 (1.68)	.001 (r = .40)	0–8+	3.20 (1.88)	3.95 (1.63)	.008 (r = .33)
Water	0–8+	5.65 (2.09)	6.06 (1.97)	.088 (r = .21)	0–8+	4.63 (2.19)	5.70 (1.75)	< .001 (r = .46)
Sweetened/fizzy drinks	0–8+	.89 (1.17)	.84 (1.39)	.792 (r = .03)	0–8+	.72 (1.22)	.62 (1.21)	.557 (r = .08)
High fat/sugar foods	0–8+	1.79 (.62)	1.55 (1.38)	.310 (r = .13)	0–8+	1.43 (1.51)	1.18 (1.11)	.212 (r = .16)
**Fruit and Veg**		15 (24.4%)	27 (39.7%)	.019		18 (29.0%)	22 (32.8%)	.629
**Physical activity**		54 (78.3%)	54 (78.3%)	1.00		30 (44.1%)	32 (46.4%)	.824

Total scales in bold and single items in normal font. Single items reported when analysis did not support a total scale score. Fruit and vegetables and physical activity values represent frequency with percentages in brackets achieving recommended amount. *P*-value results represent pre-post time effects, using paired-sample t-tests for food intake and McNemar’s tests for fruit and veg and physical activity (criteria for significance, *p* < .01). Effect size represents Pearsons r (criteria: 0.1 = small; 0.3 = medium; 0.5 = large)

### Qualitative feedback – parents

This section considers the experiences of parents, with parent quotes in Table 3 labelled as either parent H (Haringey parents) or parent E (Ealing parents). Parents could recall visiting stalls, talks and speaking with volunteers at outreach events, and heard about HENRY through baby groups, friends, children’s centres, and primary schools. Parents spoke of spending more family time together and increased enjoyment of family life. Parents developed their understanding of children’s behaviour and ways of responding to children’s feelings while remaining appropriately in charge. Parents also reported that the relationship skills they gained from the programme changed their own behaviour and helped their wider relationships. These changes were achieved through setting realistic goals, greater awareness of family eating habits, new parenting strategies, and the rapport and support from the volunteers. The programme encouraged parents to identify practical solutions and try out parenting strategies to build cooperation and a positive atmosphere, such as giving children some control by offering a choice between healthy alternatives and use of family rewards for praise and encouragement. Parents felt they had rapport with volunteers, and they were understood in a non-judgemental way. The simplicity and accessibility of the supporting materials was also appreciated.

**Table 3:** Feedback from focus groups with parents

Theme	Subtheme	Comments
**Motivation to engage**	*Getting involved with HENRY*	‘Because I was thinking, “She’s eating a little bit.” I’m thinking, “Is she full? Is she not full? Oh my God, am I starving her?”’ (Parent H) ‘He is a fussy eater and the easiest way is to distract him and just feed him, which is why I wanted HENRY, because I wanted a healthier approach for how to actually get him to sit down at the table with us and actually eat. So, yes, that’s what I expected.’ (Parent H)
**Changes made**	*Time spent with family members*	‘Time for everybody, together or alone it’s very important …. They are playing and telling stories and focus attention to them more than before.’ (Parent E) ‘But before it was all like I need to do this. I need to do that. So it was, basically, me doing everything by myself. But now I don’t feel like that. I feel like I just need to have a healthy lifestyle and my family joins in with me instead of me thinking that I have to be separate to them and do like a separate diet, do a separate gym activity.’ (Parent H)
	*Changes within the family*	‘The children would always sit at a little table and the parents would always eat separately after the children were in bed. The children would eat at, sort of 5:00 pm, I guess, and then the parents wouldn’t get home until 7:00 pm, and then they’d eat about 8:00 pm when the children were in bed. But because I was now cooking and eating with them, then we moved to the big table.’ (Parent E) ‘Yes, because I used to put hers separately, give her breakfast on her own or give her dinner on her own or feed her first and then us. But now I just put her with us, if she makes a mess, she makes a mess.’ (Parent E)
**Mechanisms of change**	*Goal setting*	‘And I kept having these really high targets for myself, saying, “I’m going to do this every week … And I wouldn’t get- it’s not possible to do it. And then I will fail. And then it will just make me feel miserable”’ (Parent H) ‘Because then I set myself targets and say, “I’m going to go to the gym five times a week. I’m going to do this.” Then it won’t work. So now, I say, “Okay, three times, four times a week I’ll go to the park. I’ll do some extra walking, instead of eating takeaways, I’ll make some food at home so the baby eats with me.”’ (Parent H)
	*Reflecting on their family’s eating habits*	‘For us, it was very useful, because it helped us to look more in depth at our family life and his activities, his eating, his routines.’ (Parent H) ‘… portion sizes. Because I’m really bad for myself. And for children, thinking how much they can eat, how big their stomachs are. I still get confused, but it’s a good reference tool to have.’ (Parent E) ‘… and labelling in the foods that’s very important. I used to do it, but after that I tried to read every fact and everything from the labelling. It’s very, very important.’ (Parent E)
	*Practical parenting strategies*	‘So I remember one session, I found it a bit silly at the time, but it helped. We were sitting at the table, me and my daughter and then we had a teddy bear there as well. And I was like, “Right, you need to give out the food on the table. Today, you be in charge.”’ (Parent H) “the children really liked their reward scheme, so we did a tree so they would draw a new leaf on the tree if they did something really helpful or extraordinary. And then mum and dad got involved as well so there was a sort of family rule tree and we could all draw a leaf if everybody thought we’d done something particularly kind or … Which worked quite well.’” (Parent E)
	*Volunteers*	‘Yes, she’s very talented in the way that she was not even vaguely judgmental. You didn’t feel like she was coming to tell you how to do something. She was just helping and suggesting.’ (Parent E) ‘But it was good, because she didn’t give up bless her. She was very persistent. I think that was a good thing. Because if it was down to me I’d probably like, “Well, forget it. I’m not going to complete it.” But she didn’t give up.’ (Parent H)
**Programme feedback**	*Programme feedback*	‘So, for me, the way it worked was that HENRY was in the book, it was nice they put in different sections, so every week there was something else we were talking about. So I think I found that very helpful. And it wasn’t very intrusive. They didn’t intervene. Just very simple, basic to a point, nice.’ (Parent H) ‘I think it should be promoted more, because it’s not really known out there for a lot of people. Not a lot of families know about it, not a lot of families hear about it.’ (Parent H) ‘And so it would have been good to do some practical activities like cook a meal together or sit down and eat a meal together.’ (Parent E)

### Volunteer characteristics and ratings

Volunteers were mostly female (94%) and aged on average 43 years old (range 26–63 years). There was a wide range of ethnicities with ‘white other’ the most prevalent (39%), which included Dutch, Spanish, French, Italian, Slovakian, and American. The volunteers were mostly employed with a high level of education on average (Bachelors, 56%), and 11 out of 18 volunteers had previous volunteer experience. Volunteers rated the training as mostly very good (72%), were moderately (47%) or very confident (53%) to deliver the programme following the training and would all definitely recommend the training to other volunteers (100%). The volunteers also rated the further support in terms of practice development groups as very helpful (67%), and individual supervision as very helpful (72%). Further ratings from volunteers showed that they were quite happy (50%) or very happy (50%) with how they delivered the programme and thought parents found the sessions very helpful (72%).

### Qualitative feedback – volunteers

This section considers the experiences of volunteers, with quotes in Table 4. When HENRY was presented to volunteers as a possibility, they responded positively because it looked interesting and they generally had a predisposition to volunteering for this type of project. Life experience was thought to be a valuable contribution to working with parents and they felt they had the freedom to incorporate this into the programme. The volunteers praised the training and thought a key feature of the programme was the holistic approach which was not just about eating. The volunteers were trained not to be prescriptive but found this quite difficult in practice at times. The volunteers were in agreement that the depth of emotions parents were dealing with were challenging and that the training could, in future, provide additional tools to deal with this.

**Table 4:** Feedback from focus groups with volunteers

Theme	Subtheme	Comments
**Engaging with HENRY**	*Volunteers’ background*	‘Me, I’ve always volunteered in the community, with children and young people, so I wanted to go onto paid work, I thought through it I could do volunteering.’ ‘I had done parenting course for my children when they were younger and whatever trainings I could get my hands on, I loved doing them. I also did an NHS Breast Feeding Support worker, so I had a little bit of a background before I came and did this training.’
	*Life experience*	‘… sometimes the parent could be going through such a difficult time and maybe your life experience as C (volunteer) has said, that you can sort of easily relate to it …, so you can relate to them.’
**HENRY training**	*Impact on volunteers*	‘Yes, it was a role-play, which was really, really interesting because we thought now we have to act as volunteers. And it really made us think, about what it’s going to be like...’ ‘I could use the techniques at home as well, with my children. So, that was quite useful for me.’
	*Not telling parents what to do*	‘And you know what the answer is, but you can’t tell them, so you have to let the parent figure it out themselves. I think that is a challenge as well, definitely.’
	*What was missing from the training?*	‘I think that was good, because it wasn’t like were advising them, you were giving them options and at the end of the day, it was their choice.’ ‘When people join, I think we should be clear, “This is not what we offer, we are just here for HENRY”. Because most of them that I’ve met, always ask me for something else, other than just the programme. It could be housing, it could be immigration.’
**Delivering HENRY**	*Materials*	‘Especially when you start off, and you don’t know how to deal with the situation, it was very helpful but it does take preparation time as well. You can’t just walk in and say “I’ll deliver the programme now” chapter one or two or whatever.’
	*Volunteer role*	‘So although, you’re giving them the toolkit and everything, they get a little bit worried about what else you’re there for.’ ‘I think people worry about social services, child protection, things like that and … They might be wondering what it is … what am I going to do with the information.’
	*Responding to parents’ needs*	‘I think maybe highlighting what is important really to the family … what I found is that one family had no issues around diet and things like that, it was more around behaviour and the other family it was more around the diet.’ ‘Sometimes you can plan your session for that day, or that week, but the only thing is, when we sometimes get there, the parents actually want to discuss something else. Then you sort of have to engage with them and listen to them first, and then you try and sort of bring them back to the session that you’ve prepared. That can be sometimes difficult, because we only have an hour to spend with them and you’re trying to plan everything in one hour, it normally just spills over that one hour I find.’
	*Challenges*	‘If you think about eating well, if there is not a proper roof over your head, or you have some kind of a problem with your health, you can’t really get everything going …’ ‘Because when you leave the family, you want to walk away having a sense of achievement that you’ve actually helped this family and supported them and now, they’re in a bit better position to help themselves. Then when it sort of breaks halfway, you don’t know whether they’re going to be alright.’
**Challenges**	*Feedback from parents*	‘I think you just get the feedback from the mums when you finish. They’re so happy or willing to tell you how it’s gone. Trying to focus on oneself, which a mum doesn’t do, the me-time and boosting the batteries. They get sort of like “Oh my God, yes”. I mean, we didn’t realise it as mothers, but now we think “Okay I do need me time. I should have down time” that sort of thing.’ ‘Because last term, there was a mum who had a child overweight, a boy, and also not eating. He only wanted takeaways, chicken nuggets, etc. and so, when we started the programme, we were trying to engage her into healthy eating etc. She knew about it, but behaviour was not very good. I only found out this term, that that boy had lost weight, he’s eating properly, engaging in school meals and also his literacy, numeracy skills have gone up.’
	*Benefits of HENRY*	‘… when you first have a child, and if there was only a book that could just help me. And for me, even though my children are adults, I really look at the whole training and think “Wow if that was around.” ‘I think sometimes just having somebody who has a bit of life experience, or from a book, that that’s what they need and I think it’s quite empowering actually.’

The volunteers had positive views about the materials, both in terms of the content and how to deliver it. They particularly found these useful at the beginning when supporting parents. Even though materials were viewed positively the sessions still took preparation time. When asked to compare the amount of time exploring parenting issues and strategies as opposed to lifestyle issues, the volunteers reflected the need to balance standardisation of delivery with tailoring for individual parents. Parents had a range of challenges and not just those related to family lifestyle and volunteers felt they had succeeded when they received positive feedback from families. The volunteers’ thought the programme was effective and that they would have appreciated having access to it when their own children were small.

## Discussion

This study reported a mixed-methods evaluation exploring the feasibility of delivering a HENRY programme by volunteers to parents with children up to 5 years old. The programme managed to engage parents from a variety of ethnic backgrounds, who were often not working and living on a low household income but were well educated. Feasibility was demonstrated by parents attending the majority of programme sessions, rating the programme as very useful, and a willingness to recommend it to others. Improvements were observed in reported parent and child emotional wellbeing, parenting efficacy, fruit and vegetable consumption, and several family eating and food purchasing behaviours. Parents reported not being concerned that volunteers were delivering the programme and tended to be motivated to engage because of perceived eating issues, including children not eating. Perceptions of children being reluctant to try new foods and being ‘picky’ are well-known barriers for parents aiming to instil healthy dietary behaviours [34]. The dietary input was well-received and all participants could recall information which they considered helpful. They also thought the behavioural element was integral to how the programme worked. Another important aspect of the programme was the relationships that were built with volunteers. This was shown to be crucial in another qualitative evaluation of a group-based child weight management intervention [35].

The feasibility of recruiting and training volunteers was shown through positive ratings of the training and volunteer’s confidence and happiness in delivering the sessions, and appreciation of the ongoing development groups and supervision available. The volunteers were from a wide range of ethnic backgrounds, well educated, and had relevant experience that allowed them to work effectively with parents. There is a possibility that the richness of volunteers available in north and west London may be greater than in other areas of the UK. Other locations with universities offering nutrition or dietetics and social work training may also provide well educated volunteers. However, recruiting such high calibre volunteers may be an issue in areas without higher education institutes. HENRY appears to have provided a beneficial vehicle for volunteers’ practical experiences, either as part of their degrees or hands-on experience for those wanting to initiate careers in these fields. Non-student volunteers also reported that having volunteering for HENRY on their CVs was a useful step into full-time employment. The personal benefits that volunteers perceived, as well as their enjoyment of being trained and then working with parents, is likely to encourage high calibre volunteers and these findings may be useful in future recruitment drives.

There is an ongoing need for programmes which can address knowledge and efficacy, particularly in low-income parents, to alleviate the rising levels of overweight [1] and inactive [2] pre-school children. Programmes targeting child weight-related outcomes such as BMI have generally been unsuccessful [3]. However, community-based approaches to improve parent nutrition education and eating behaviour have shown more positive results [5, 8, 9], and can comprise part of successful population-level approaches to reduce obesity [17]. Evaluations of HENRY have focused on parent-targeted programme delivery by either HENRY staff or HENRY-trained staff working in local public health teams, health visiting services, and children’s centres [11–16]. This study attempted to fill a gap in the evidence by assessing the feasibility of parent-targeted programmes in community venues or parents’ homes using volunteers.

There are several potential benefits of involving volunteers in this type of programme. First, given consistent reductions in public health budgets, novel ways of delivering evidence-based support to parents with young children could be helpful in sustaining high quality health promotion. Second, volunteering can provide personal benefits to wellbeing, depression, life satisfaction, and even decreased mortality [22]. Third, volunteering can develop social capital in terms of improving community relationships, and learning and motivation for volunteers to impact social change [36]. Fourth, having volunteer health workers embedded in the communities in which they deliver programmes can be a crucial community resource [37, 38]. Future research should examine whether utilizing volunteers is sustainable in terms of turnover due to lack of remuneration [37] and the utility of training and experience for volunteers’ future career aspirations.

This study was novel in attempting to both quantitatively and qualitatively evaluate the feasibility of a programme delivered by trained volunteers from similar locations and ethnic backgrounds to programme participants. This study achieved results in line with previous evaluations apart from reported physical activity. Levels of physical activity were very high at baseline with 78% of parents reporting achieving the recommended amount, which compares favourably to the general public (59% for females) [39] and parents from previous evaluations (e.g. 67%) [15]. It may be that this was an active sample with little room for improvement on this outcome.

As this was a low-cost service evaluation assessing feasibility, quantitative limitations must be acknowledged. These include the modest total sample size, the psychometric properties of some measures (that failed to achieve adequate internal consistency) and a lack of research capacity for richer dietary assessment. Self-report measures are used widely but they are liable to response bias and it is possible that this impacted upon the findings by inflating responses. Therefore, caution is warranted when interpreting the results, particularly those concerning dietary intake and physical activity. Nevertheless, it is perhaps worth noting that while this methodology does not measure actual behaviour change, the changes observed at least indicate that healthy lifestyle messages were being recognised. We would also argue that self-report measures were warranted in this study: more rigorous measures of food intake, such as weighing of portions, would have been impractical and may well have been off-putting, potentially reducing participation. Critically, they would also have impacted upon the intervention itself, which is founded on a non-prescriptive approach and encourages self-determination of goals. In addition, although the programme had several procedures in place to maximise fidelity, this was not formally assessed. Furthermore, there was no control group and, so despite corroborating feedback from parents, it is difficult to attribute the observed improvements directly to the programme, or to assess which components were most effective.

## Conclusion

This study showed that it is feasible to recruit and train volunteers to deliver evidence-based obesity prevention programmes to parents in their own communities. Parents rated the programme as very useful and would recommend to others. Volunteers rated the training and support positively, felt confident delivering the programme, and would also recommend the training and experience of being a HENRY volunteer. This service evaluation provided promising data showing that this approach may be feasible, but future research needs to use randomised controlled trials comparing the HENRY approach with treatment as usual and/or alternative interventions to provide evidence that this approach is effective.

## Supplementary Information

**Additional file 1.**

## Data Availability

The datasets used and/or analysed during the current study are available from the corresponding author on reasonable request.

## Abbreviations

HENRY: Health, Exercise, and Nutrition for the Really Young
BMI: Body Mass Index
UK: United Kingdom
LE: Laurel Edmunds
SPSS: Statistical Package for the Social Sciences
IBM: International Business Machines
CV: Curriculum vitae
