# Supplementary material for: Testing the feasibility of a sustainable preschool obesity prevention approach: a mixed-methods service evaluation of a volunteer-led HENRY programme
Source: BMC Public Health. 2021 Jan 6;21:46. doi: 10.1186/s12889-020-10031-w (PMC7789777; doi:10.1186/s12889-020-10031-w)
Supplement: Supplementary file 1 — Additional file 1. [file 12889_2020_10031_MOESM1_ESM.zip › Supplementary File - HENRY focus group schedulesR3.docx]

**Focus Group Schedule for Volunteers**

**Introduction**

Explain my background and role. This focus group is about volunteers’ ideas about being part of the HENRY programme. We are are very interested in hearing about their own experiences and views, whatever they are. *Would it be OK if I record the conversations, this will just mean that I won’t have to write everything down. The recording will only be listened to by myself and will be anonymised to ensure that you cannot be identified. Also, you can leave the focus group at any time. Thank you.*

The aim is to provide themes around issues of interest (e.g. quality of the training) and also allows for novel subjects to arise (e.g. any unintended consequences). Additionally, other issues of importance to volunteers may emerge during the course of the focus groups.

**The main topics covered in the focus groups will be:**

- To investigate their experiences of delivering the programmes and working with the families, focusing on their expectations, their perceptions of parents and about the programme.
- To establish and assess how they got involved in being a volunteer for HENRY and their experiences of being recruited.
- To what extent did the training prepare them for delivering HENRY and are there changes in the training they consider would address any gaps, or would be more helpful.
- Their perceptions of the programme in terms of how they felt it came across to parents (communications, support, trust, creating relationships).
- On reflection, what were the most helpful aspects of their training and what they would do differently in delivering the programme.
- To assess any changes made by volunteers and their families who are part of the programme.

**The Guide:**

Opening questions

How many families have you supported using the HENRY programme?

Introduction Questions

- How did you find out and get involved with HENRY/Family Lives?
- What were your expectations of volunteering with Family Lives?
- What happened when someone suggested you might like to be a volunteer?
- (How were you recruited?)
- When did this happen? (timing of recruitment)?
- Could this have been done in a better way?
- What motivated you to get involved with Family Lives/HENRY as a volunteer?

The Programme

- What are your impressions of the programme?
- What did you like best about the programme?
- What didn’t you like about the programme?
- What do you think the families like about the HENRY programme?
- Where you aware of families appreciating the HENRY programme and making changes?
- To what extent and in what ways has the HENRY programme changed their lifestyles?

Delivery

- Which aspect of the programme did you enjoy delivering the most?
- What were your expectations of being a part of the delivery team?
- Were there instances when you were surprised about things that happened in the sessions?
- Were there any aspects that you found challenging or thought didn’t go well?
- Did you feel able to maintain the integrity of the programme i.e. deliver it in a way that was consistent with the training? (are you able to give an example of this?)
- Did you ever have any regrets about your involvement with the HENRY programme?
- How do you feel about continuing to deliver the HENRY programme (if applicable)?

Training

- What were your overall impressions of the training?
- What were the most useful aspects of the training?
- What did you take away as the important messages, or approaches, or ways of thinking, from the training?
- Did you experience anything while delivering the programme that you felt the training didn’t cover?

Information

- What information were you provided with concerning the HENRY/Family Lives training?
- Did you understand all the information?
- How helpful was the information?
- Did you feel there is anything missing?

Feedback

- How did you feel about the feedback you received during your training in HENRY?
- What support has been offered to you either during training or delivering the HENRY programme?

Improvements

- Do you think there is anything else that can be added to the training or the programme?

Ending Questions

- Is there anything that you would like to add?

**Focus Group Schedule for Parents**

NOTE – This semi-structured schedule covers the topics that will be included in the focus groups, but the exact wording and order will vary depending on the responses. Later focus groups are likely to include additional areas which arise from analysis of the first set of transcripts. Sensitive use of prompts are likely to encourage parents to expand their opinions on any of the topics in the schedule, looking particularly for anything that might have a story attached to it.

**Introduction**

Explain my background and role. This focus group is about parents and guardians’ ideas about taking part in the HENRY programme. We are very interested in hearing about their own experiences and views, whatever they are. *Would it be OK if I record the conversations, this will just mean that I won’t have to write everything down. The recording will only be listened to by myself and will be anonymised to ensure that you cannot be identified. Also, you can leave the focus group at any time. Thank you.* The aim is to provide themes around issues of interest (e.g. impact of the intervention) and also allows for novel subjects to arise (e.g. any unintended consequences). Additionally, other issues of importance to parents and children may emerge during the course of the focus groups.

**The main topics covered in the focus groups will be:**

- To what extent and in what ways has the HENRY programme changed their lifestyles (comparing before, during and after, focusing on their expectations of the intervention and their reflections of taking part and how their lives are now)?
- To what extent and in what ways did the programme change the knowledge, attitudes and confidence within families with regard to healthy lifestyles? These questions will likely focus on parenting, dietary and physical activity changes and may include understanding of family function.
- How did the people delivering the programme help, communicate, instil trust, create relationships with the families?
- On reflection, what parts were most helpful and what messages would participants like to pass on to programme deliverers, other parents? This is likely to pull out the main areas where the programme made a difference and how they think others may benefit.
- The opportunity will then arise to compare these findings with other outcome measures if appropriate. The guide below indicates areas of interest rather than a script.

**The Guide:**

Opening questions

- Are your children boys or girls? (with responses being shared around the group in turn)
- What was the best thing about the HENRY programme?

Introduction questions

- How did you find out and get involved with HENRY?
- What were your expectations of HENRY?
- What were your early experiences of HENRY?
- What motivated you to get involved with HENRY?
- What could be done to promote the programme and encourage other families to attend?

The Programme

- What did you like best about the programme activities?
- What didn’t you like about the programme activities?
- What did your child like?
- What did you think about the parenting part of HENRY?
- Overall was the programme what you expected?
- What changes have you made at home with your family since attending the HENRY programme?
- And are you still implementing the changes?
- How difficult has it been to make these changes?
- What helped?
- What impact would you say it had on your child/children/family?

HENRY Personnel

- What did you think of the people who ran the programme?
- Can you tell me about how they communicated with you?
- What sort of atmosphere did they create?
- What were the good points about how they worked with you?
- Were they any downsides to how they worked with you?

Information

- What information were you provided concerning HENRY?
- Did you understand all the information?
- How helpful was the information?
- Did you feel there is anything missing?

Feedback

- How did you feel about the feedback concerning your progress on the HENRY programme?
- What support has been offered to you either during or since leaving the HENRY programme?
- Have you told anyone else about HENRY?
- What difference has the HENRY programme made for you and your family?
- How have others (partners and relatives) responded to your being part of the HENRY programme?

Improvements

- How has the HENRY programme helped you and your child?

Ending Questions

- Is there anything that you would like to add?
